# MMP-12 Deficiency Attenuates Angiotensin II-Induced Vascular Injury, M2 Macrophage Accumulation, and Skin and Heart Fibrosis

**DOI:** 10.1371/journal.pone.0109763

**Published:** 2014-10-10

**Authors:** Lukasz Stawski, Paul Haines, Alan Fine, Lidia Rudnicka, Maria Trojanowska

**Affiliations:** 1 Arthritis Center, Boston University School of Medicine, Boston, Massachusetts, United States of America; 2 Pulmonary Center, Boston University School of Medicine, Boston, Massachusetts, United States of America; 3 Faculty of Health Sciences, Medical University of Warsaw, Warsaw, Poland; 4 Department of Neuropeptides, Mossakowski Medical Research Centre Polish Academy of Sciences, Warsaw, Poland; Albert Einstein College of Medicine, United States of America

## Abstract

MMP-12, a macrophage-secreted elastase, is elevated in fibrotic diseases, including systemic sclerosis (SSc) and correlates with vasculopathy and fibrosis. The goal of this study was to investigate the role of MMP-12 in cardiac and cutaneous fibrosis induced by angiotensin II infusion. Ang II-induced heart and skin fibrosis was accompanied by a marked increase of vascular injury markers, including vWF, Thrombospondin-1 (TSP-1) and MMP-12, as well as increased number of PDGFRβ^+^ cells. Furthermore Ang II infusion led to an accumulation of macrophages (Mac3^+^) in the skin and in the perivascular and interstitial fibrotic regions of the heart. However, alternatively activated (Arg 1^+^) macrophages were mainly present in the Ang II infused mice and were localized to the perivascular heart regions and to the skin, but were not detected in the interstitial heart regions. Elevated expression of MMP-12 was primarily found in macrophages and endothelial cells (CD31^+^) cells, but MMP-12 was not expressed in the collagen producing cells. MMP-12 deficient mice (MMP12KO) showed markedly reduced expression of vWF, TSP1, and PDGFRβ around vessels and attenuation of dermal fibrosis, as well as the perivascular fibrosis in the heart. However, MMP-12 deficiency did not affect interstitial heart fibrosis, suggesting a heterogeneous nature of the fibrotic response in the heart. Furthermore, MMP-12 deficiency almost completely prevented accumulation of Arg 1^+^ cells, whereas the number of Mac3^+^ cells was partially reduced. Moreover production of profibrotic mediators such as PDGFBB, TGFβ1 and pSMAD2 in the skin and perivascular regions of the heart was also inhibited. Together, the results of this study show a close correlation between vascular injury markers, Arg 1^+^ macrophage accumulation and fibrosis and suggest an important role of MMP-12 in regulating these processes.

## Introduction

Systemic sclerosis (SSc) is a complex autoimmune disorder of unknown etiology characterized by vascular alterations, activation of the immune system and fibrosis of the skin and internal organs [1], [2]. Endothelial cell damage manifests early in the disease as evidenced by elevated levels of the characteristic vascular injury markers such as von Willebrand Factor (vWF) and Thrombospondin 1 (TSP-1) [3], [4]. Furthermore, elevated mRNA levels of TSP-1 correlate with modified Rodnan skin score, suggesting a link between vascular injury and fibrosis in SSc patients [5]. Endothelial cell injury is not limited to the skin, but also affects other organs, including heart, GI tract, kidneys, lungs and central nervous system [6], [7]. The main effector cells in SSc responsible for the fibroproliferative process are activated local myofibroblasts, a unique population of mesenchymal cells, which produce excessive amounts of extracellular matrix (ECM) proteins resulting in widespread tissue fibrosis. However, the origin of these ECM-producing fibroblasts has not been completely elucidated [8], [9], [10]. While pathways contributing to activation of fibroblasts have been widely investigated, the mechanisms that contribute to vessel degeneration in SSc are still poorly understood. What's more, it is unclear how the initial vascular dysfunction is connected to the activation of fibroblasts and progression of skin and organ fibrosis that is the hallmark of this disease.

Angiotensin II (Ang II), a main component of the renin–angiotensin system (RAS), is a vasoactive peptide that regulates vascular constriction, salt and water retention, and increases blood pressure [11]. Ang II was also reported to cause endothelial cell injury by increasing the production of reactive oxygen species (ROS) [12], [13], [14], as well as inducing ER stress [15] and endothelial cell apoptosis [15], [16]. In addition, Ang II is a potent profibrotic molecule that induces kidney, liver, heart and skin fibrosis [17], [18], [19], [20]. Ang II, through its receptors AT1 and AT2, activates profibrotic TGFβ signaling pathways, but also induces expression of proinflammatory mediators, such as monocyte chemoattractant protein-1 (MCP-1) [18], [21]. Previous studies indicate that Ang II may be involved in the pathogenesis of SSc [22]–[23], and Ang II blockade has been traditionally used as a vasodilator therapy in renal, pulmonary and cardiac complications in SSc patients [24], [25], [26].

The matrix metalloproteinase (MMP) family of zinc dependent proteases and their tissue inhibitors (TIMPs) are known to control extracellular matrix (ECM) homeostasis and play an important role in the physiological processes during development and morphogenesis as well as in pathological processes including atherosclerosis, cancers, heart and skin diseases [27]. Matrix metallopeptidase 12 (MMP-12), also known as macrophage elastase, has broad substrate specificity for extracellular components and was shown to be a key player in tissue remodeling associated with many pathological conditions such as chronic inflammation and fibrosis [27]. In fact, MMP-12 deficiency resulted in decreased inflammation and collagen deposition in Fas-L [28] and bleomycin [29] -induced lung fibrosis. Contrary to these findings, in a different study MMP12KO mice showed no significant change in inflammation and ECM production in response to bleomycin treatment [30]. Likewise, MMP-12 deficient mice were not protected from the development of IPS (Idiopathic Pneumonia Syndrome) after bone marrow transplant, and in addition developed fibrosis associated with increased expression of integrin-β6, a potent activator of transforming growth factor (TGF)-β [31]. Importantly, MMP-12 is highly elevated in the serum and connective tissue of SSc patients and correlates with vascular damage and the severity of skin and pulmonary fibrosis [32]. However, the pathological role of MMP-12 has not been assessed in a mouse model of scleroderma associated with vascular impairment. Given the potential involvement of MMP-12 in both vascular injury and fibrosis in the pathogenesis of SSc, the goal of this study was to investigate the contribution of MMP-12 to these processes using the Ang II model of skin and heart fibrosis.

## Methods

### Subcutaneous infusion of Angiotensin II using ALZET osmotic mini-pumps

MMP12KO and C57BL/6 mice were purchased from The Jackson Laboratory. All of the experiments were performed under the guidelines of the Boston University Institutional Animal Care and Use Committee. Alzet osmotic miniature pumps (model 2002) delivering Angiotensin II (Sigma-Aldrich, St. Louis, MO) at a rate of 1000ng/kg/min (pressor dose) or PBS, were implanted subcutaneously on the back of 12-week old mice. After 14 days mice were sacrificed and the heart and the skin surrounding the pump outlet was collected.

This study was carried out in strict accordance with the recommendations in the Guide for the Care and Use of Laboratory Animals of the National Institutes of Health. The protocol was approved by the Committee on the Ethics of Animal Experiments of the Boston University (Permit Number: AN-15037.2013.10). All surgery was performed under anesthesia, and all efforts were made to minimize suffering.

### Gomori's Trichrome staining

Gomori's Trichrome staining was used to detect collagen fibers and collagen deposition in the mouse skin. The skin samples were fixed in 4% paraformaldehyde for 24 h and then processed for paraffin embedding. Staining was performed on 8 µm thick paraffin sections following the manufacturer's instructions (Chromaview, Dublin, OH, Gomori's Trichrome Blue Collagen Kit cat#: S7440-19). Collagen fibers were stained blue, nuclei were stained black, and the background was stained red.

### Picrosirius Red staining

Picrosirius Red staining was used to detect collagen deposition in the mouse heart. The heart samples were fixed in 4% paraformaldehyde for 24 h and then processed for paraffin embedding. Staining was performed on 8 µm thick sections. Briefly, sections were mounted on APES (aminopropyltriethoxy silane solution)-coated slides, deparaffinized with Histo-Clear (National Diagnostics, Atlanta, GA), and rehydrated through a graded series of ethanol. Subsequently sections were incubated for 1 hour in pre-warmed Bouin's solution (Thermo Scientific, Rockford, IL) at 55°C followed by 10 min staining with 0.1% Fast green (Fisher Scientific, Pittsburgh, PA) and 30 min staining with 0.1% Sirius Red (Sigma-Aldrich, St. Louis, MO) in saturated picric acid. Then sections were rinsed in 1% acetic acid, dehydrated, mounted and examined with an Olympus BH-2 microscope (BH-2; Olympus, Center Valley, PA).

### Hydroxyproline assay

Collagen deposition was quantified by measuring total hydroxyproline content in 4 mm skin punch biopsies obtained from PBS and Ang II infusion sites using a previously described method with some modifications [33]. Briefly, the skin samples were hydrolyzed with 6 M sodium hydroxide at 110°C for 12 h. The hydrolyzate was then oxidized with oxidation buffer (one part 7% chloramine T and four parts acetate citrate buffer) for 4 min at room temperature. Ehrlich's aldehyde reagent was added to each sample, and the chromophore was developed by incubating the samples at 65°C for 25 min. Absorbance of each sample was read at 560 nm using a spectrophometer. Results were expressed as total hydroxyproline content (µg) per 0.1 g of tissue. A standard curve was performed for all hydroxyproline measurements using known quantities of hydroxyproline.

### Immunofluorescence staining on frozen sections

For all immunofluorescence stainings heart and skin samples were directly embedded in O.C.T. compound, flash frozen, and stored at −80°C. Staining was performed on 8 mm cryosections. Double immunofluorescent staining on mouse tissue using mouse monoclonal antibodies and sequential incubation with primary antibodies was performed according the protocol described below. Briefly, slides were blocked with a BLOXALL endogenous peroxidase and alkaline phosphatase blocking solution (Vector Laboratories, Burlingame, CA) for 20 min. After washing, tissue sections were incubated with unconjugated Fab fragment donkey-anti-mouse (Jackson ImmunoResearch, West Grove, PA) 0.1 mg/ml in PBS at RT for 1 h. Tissue sections were then washed and incubated with primary Ab to mouse monoclonal MMP-12 (Santa Cruz, CA) diluted 1∶100 in PBS at 4°C for 1 h. In the next step, sections were washed and incubated with secondary antibody biotynilated Fab fragment donkey-anti-mouse (Jackson ImmunoResearch, West Grove, PA) diluted 1∶400 in PBS at RT for 30 min. This was followed with an Alexa Fluor 594-labeled Streptavidin (Invitrogen, Carlsbad, CA) diluted 1∶500 for 1 h at RT. In the second blocking step slides were incubated with 3% BSA (Sigma-Aldrich, St. Louis, MO) in PBS for 1 h at RT. Tissue sections were then washed and incubated with second primary antibodies: rat anti-Mac3 (BD Pharmingen, San Diego, CA) diluted 1∶100 or rabbit polyclonal anti-mouse Collagen type I (GeneTex, Irvine, CA) diluted 1∶100 at 4°C overnight. In the last step slides were incubated with Alexa Fluor 488 anti-rat (Invitrogen; Carlsbad, CA) or Alexa Fluor 488 anti-rabbit (Invitrogen; Carlsbad, CA) diluted 1∶500 for 1 h at RT. Coverslips were mounted using Vectashield with DAPI (Vector Laboratories, Burlingame, CA) and staining was examined using a FluoView FV10i confocal microscope system (Olympus, Center Valley, PA) at 488 nm (green), 594 nm (red) and 405 nm (blue).

### Immunohistochemistry

Immunohistochemistry was performed on formalin-fixed, paraffin-embedded 8 µm skin tissue sections using the Vectastain ABC kit (Vector Laboratories, Burlingame, CA) and Vector ImmPress Rabbit-AP kit according to the manufacturer's instructions. Briefly, sections (5 µm thick) were mounted on APES (aminopropyltriethoxy silane solution)-coated slides, deparaffinized with Histo-Clear (National Diagnostics, Atlanta, GA), and rehydrated through a graded series of ethanol. Endogenous peroxidase was blocked by incubation in 3% hydrogen peroxide for 30 minutes, followed by incubation with 0.15 M glycine for 45 min., and normal blocking serum for 1 hour. For single immunostaining sections were incubated overnight at 4°C with antibodies against vWF (Polyclonal Rabbit Anti-Human Von Willebrand Factor, DAKO, Carpinteria, CA), TSP-1 (mouse monoclonal anti-Thrombospondin 1, Novus Biologicals, Littleton, CO), MMP-12 (rabbit polyclonal anti-MMP-12, Abcam, Cambridge, MA), PDGFRβ (rabbit polyclonal anti-PDGFRβ, Cell Signaling, Danvers, MA), Arg1 (rabbit polyclonal anti-Arginase 1, LifeSpan Biosciences, Seattle, WA) PDGFBB (rabbit polyclonal anti-PDGFBB, Abcam, Cambridge, MA), TGFβ1 (mouse monoclonal anti-TGFβ1, Abcam, Cambridge, MA) and pSMAD2 (rabbit polyclonal anti-pSMAD2, Cell Signaling, Danvers, MA) diluted 1∶100 in blocking buffer, followed by incubation for 30 minutes with a biotinylated secondary antibody solution. A solution containing avidin∶biotin∶peroxidase complexes was applied to the sections subsequently. Immunoreactivity was visualized with diaminobenzidine (Vector Laboratories, Burlingame, CA), and the sections were counterstained with hematoxylin. Double immunostaining on mouse tissue using mouse monoclonal antibodies and sequential incubation with primary antibodies was performed according the protocol described below. Briefly, slides were incubated with unconjugated Fab fragment donkey-anti-mouse (Jackson ImmunoResearch, West Grove, PA) 0.1 mg/ml in PBS at RT for 1 h. Tissue sections were then washed and incubated with primary Ab to mouse monoclonal MMP-12 (Santa Cruz, CA) diluted 1∶100 in PBS at 4°C for 1 h. Next, sections were washed and incubated with biotinylated Fab fragment donkey-anti-mouse secondary antibody (Jackson ImmunoResearch, West Grove, PA) diluted 1∶400 in PBS at RT for 30 min. The first color was developed using a solution containing avidin∶biotin∶peroxidase complexes. Immunoreactivity was visualized with diaminobenzidine (Vector Laboratories, Burlingame, CA). In the next step sections were incubated with primary Ab rat anti-mouse CD31 (DiaNova, Hamburg, Germany) or rat anti-Mac3 (BD Pharmingen, San Diego, CA) diluted 1∶300 and 1∶100, respectively. Sections were then washed and incubated for 30 min with unconjugated Rabbit anti-Rat IgG. The second color was developed using the ImmPress Rabbit-AP kit (Vector Laboratories, Burlingame, CA). Immunoreactivity was visualized with the Vector Blue Alkine Phosphatase (AP) substrate kit (Vector Laboratories, Burlingame, CA). Images were collected using a microscope (BH-2; Olympus, Center Valley, PA).

### Western blot

Human dermal microvascular endothelial cells (HDMECs) were isolated from human foreskin as previously described [34]. Upon written informed consent and in compliance with the Institutional Review Board of Human studies, written approval was obtained from Perinatal Committee (IRB number H-29190) of Boston University Medical School. Cells were cultured on bovine collagen-coated 6-well plates in EBM medium supplemented with 10% FBS, and EC growth supplement mix at 37°C with 5% CO_2_ in air. The culture medium was changed every other day. For Western blot, whole-cell extracts were prepared from HDMECs using lysis buffer with the following composition: 1% Triton X-100, 50 mmol/L Tris-HCl (pH 7.4), 150 mmol/L NaCl, 3 mmol/L MgCl_2_, 1 mmol/L CaCl_2_, proteinase inhibitor mixture (Roche), and 1 mmol/L phenylmethyl sulfonyl fluoride. Protein extracts were subjected to SDS-PAGE and transferred to nitrocellulose membranes. Membranes were incubated overnight with primary antibody, washed, and incubated for 1 hour with secondary antibody. After washing, visualization was performed by enhanced chemiluminescence (Pierce, Rockford, IL).

### Statistical analyses

All data were analyzed by the Student's paired T-test. The level for statistical significance was set at p≤0.05.

## Results

### Ang II induces vascular injury in mouse heart and skin

**Figure 1:**
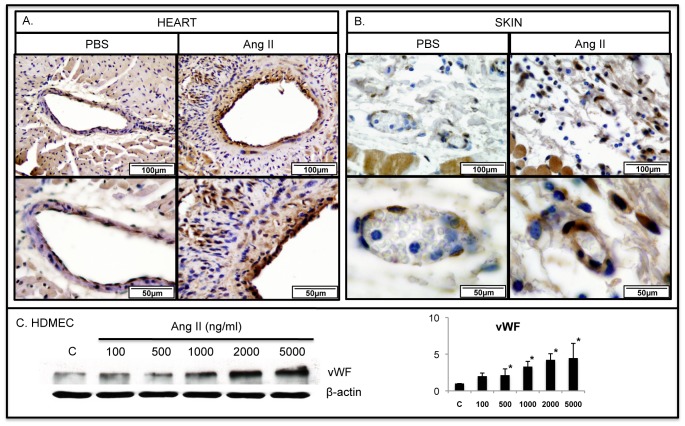
Ang II induces expression of vWF in mouse heart and skin. IHC staining of vWF was performed on paraffin sections from the heart (**A**) and skin (**B**) of PBS and Ang II treated WT mice. Representative photographs are shown from five animals per group. **C**. vWF protein levels were increased in Ang II treated HDMECs in a dose dependent manner (*p≤0.05).

To assess the effect of Ang II on the heart and skin vasculature we employed immunohistochemical staining to examine vascular injury markers: vWF, TSP-1 and MMP-12. In parallel experiments the effect of Ang II on protein levels of those markers was examined in cultured HDMECs. Heart and skin sections from PBS treated mice showed low expression of vWF in vascular endothelium and no detectable expression in perivascular cells. In contrast, heart and skin sections from Ang II treated mice showed an increased expression of vWF in endothelial cells and an increased number of vWF positive cells in the perivascular region (Fig. 1A, B). vWF protein levels were significantly induced in HDMECs after 24 hour stimulation with Ang II (Fig. 1C).

**Figure 2:**
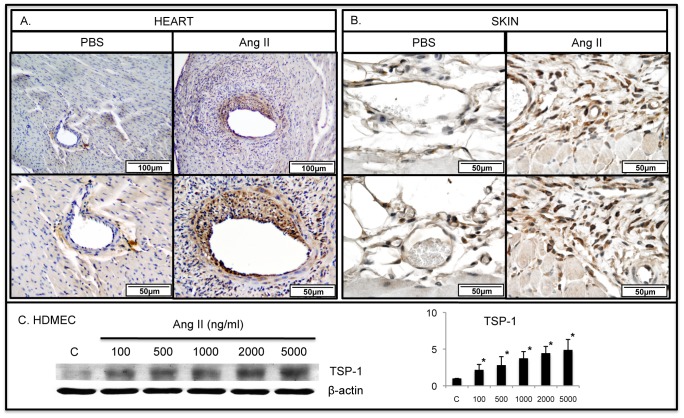
Ang II increases the number of TSP-1 positive cells in mouse heart and skin. IHC staining of TSP-1 was performed on paraffin sections from the heart (**A**) and skin (**B**) of PBS and Ang II treated WT mice. Representative photographs are shown from five animals per group. **C**. TSP-1 protein levels were increased in Ang II treated HDMECs in a dose dependent manner (*p≤0.05).

TSP-1 showed very little expression in control PBS-treated mice, however its presence was markedly increased in endothelial cells and perivascular connective tissue cells in heart and skin sections from Ang II treated mice (Fig. 2A, B). Likewise, Ang II potently induced TSP-1 expression in HDMECs (Fig. 2C).

**Figure 3:**
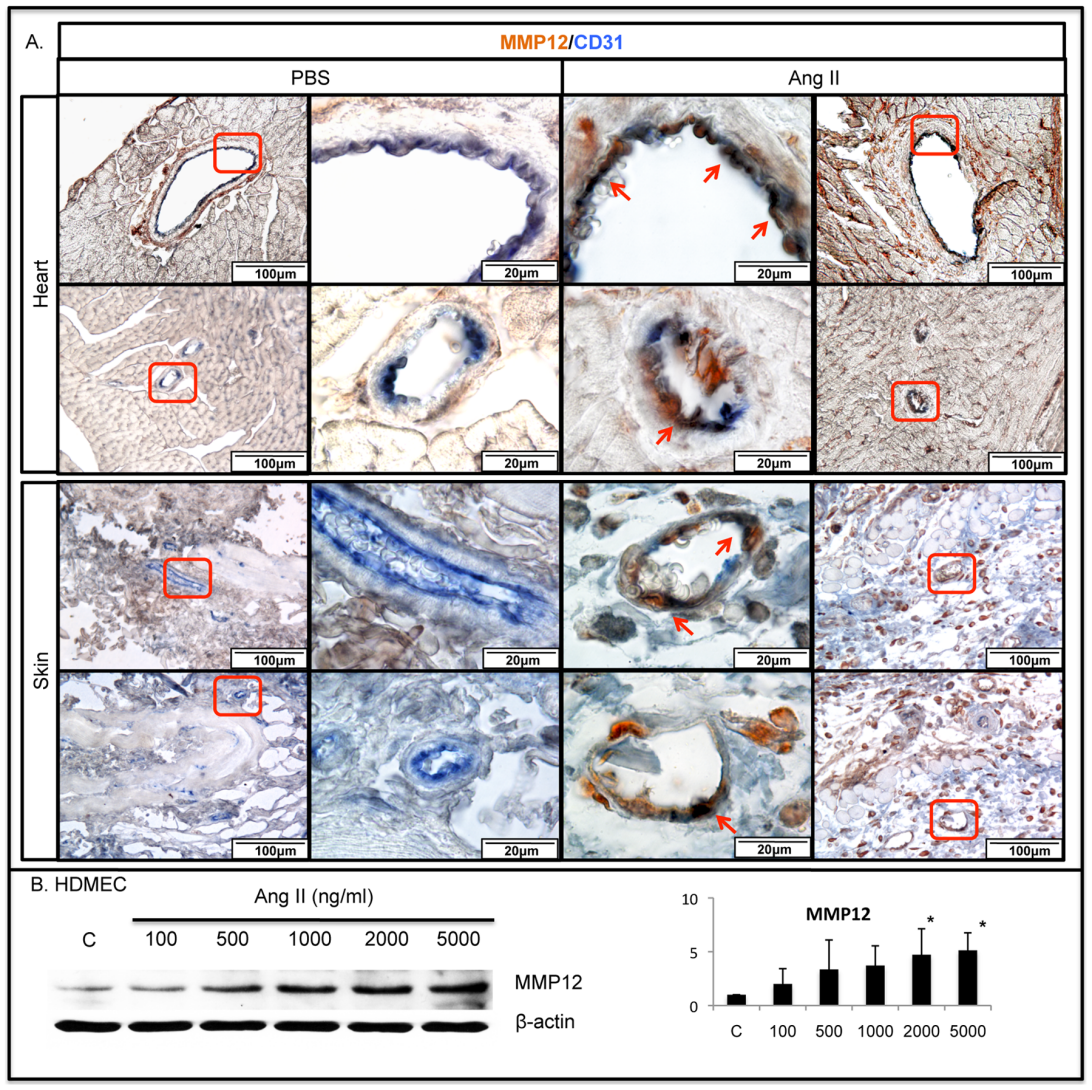
Ang II increases the number of MMP-12/CD31 positive cells in mouse heart and skin. Double IHC staining of MMP-12/CD31 was performed on paraffin sections from the heart (**A**) and skin (**B**) of PBS and Ang II treated WT mice. Representative photographs are shown from five animals per group. Arrows indicate double positive cells. **C**. MMP-12 protein levels were increased in Ang II treated HDMECs in a dose dependent manner (*p≤0.05).

We have recently reported elevated expression of MMP-12 in pulmonary endothelial cells in response to Bleomycin injection [35]. MMP-12 is a potent inhibitor of angiogenesis and was also shown to induce endothelial cell apoptosis [36], [37]. MMP-12 was only occasionally expressed in dermal cells and in peri-vascular regions in the heart in PBS treated mice. Ang II treated mice showed increased expression of MMP-12 in endothelial cells (MMP-12/CD31 double positive cells) and other perivascular cells in the heart and in the skin (Fig. 3A, B). In a corresponding experiment cultured HDMECs treated with Ang II also demonstrated increased levels of MMP-12 (Fig. 3B). Additionally, a moderate increase of apoptosis was observed in the heart and skin of Ang II infused mice (Figure S1). Together, these data are consistent with a damaging effect of Ang II on the vasculature, in part mediated through induction of anti-angiogenic mediators such as TSP-1 and MMP-12.

### Characterization of MMP-12-positive cells in the Ang II model

**Figure 4:**
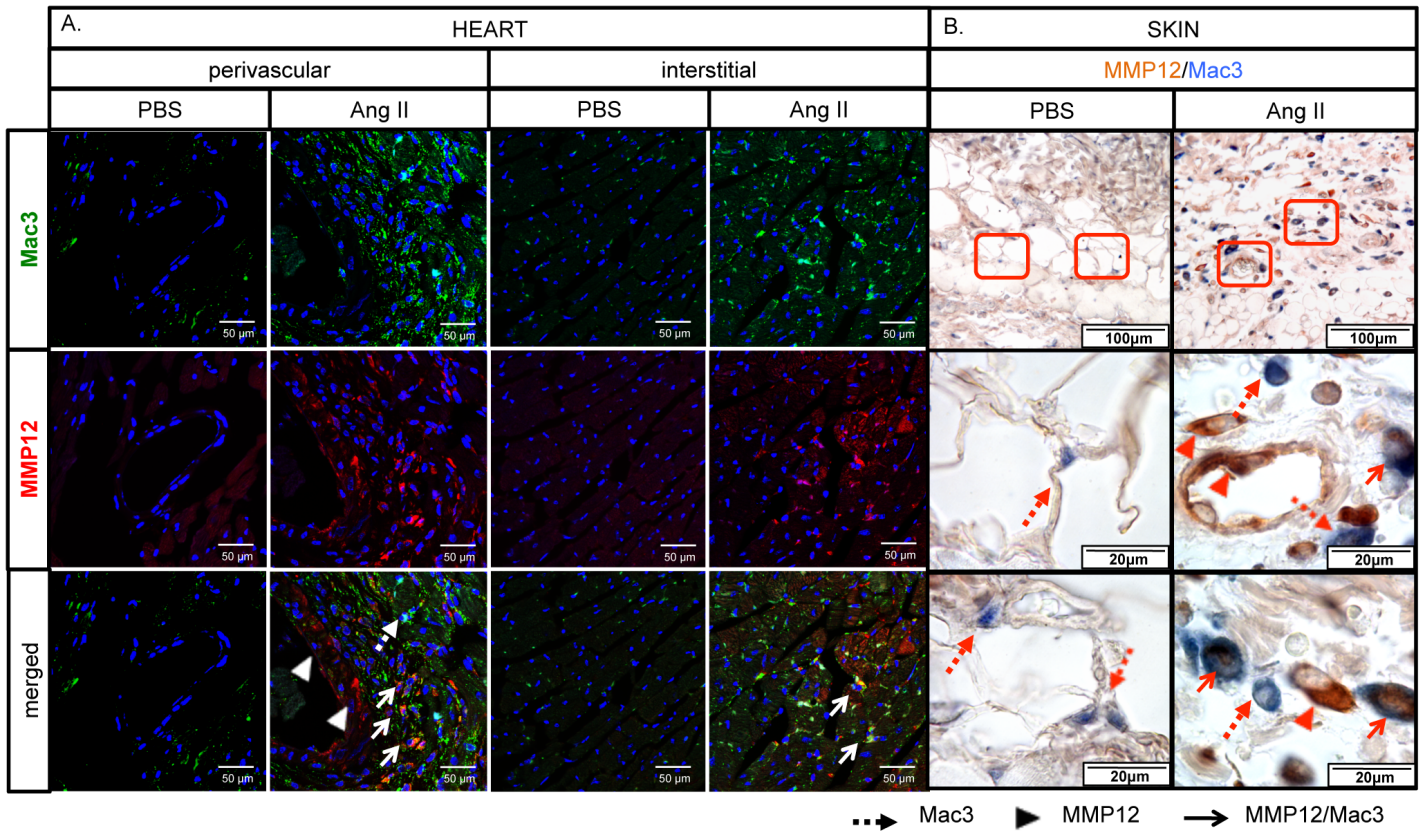
Ang II increases the number of MMP-12/Mac3 positive cells in mouse heart and skin. Double staining of MMP-12/Mac3 was performed on cryosections from the heart (**A**) and paraffin sections from the skin (**B**) of PBS and Ang II treated WT mice. Representative photographs are shown from five animals per group. Dotted arrows: Mac3 positive cells, arrowheads: MMP-12 positive cells, arrows: MMP-12/Mac3 double positive cells.

**Figure 5:**
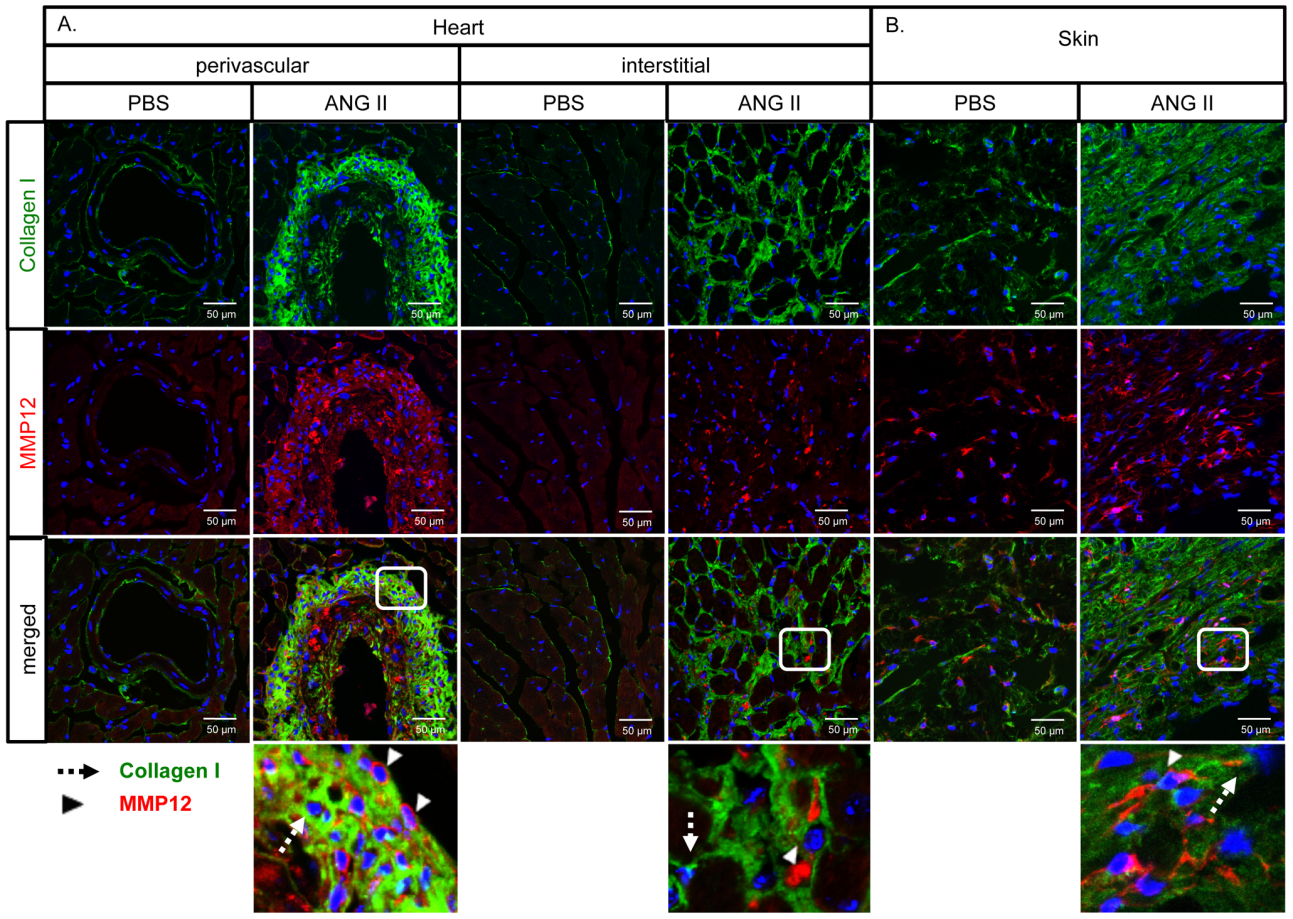
Distribution of MMP-12 and Col-I positive cells in Ang II treated mouse heart and skin. Double IF staining of MMP-12/Collagen type I was performed on cryosections from the heart (**A**) and skin (**B**) of PBS and Ang II treated WT mice. Representative photographs are shown from five animals per group. Dotted arrows: Collagen type I positive cells, arrowheads: MMP-12 positive cells.

To further characterize the cellular origin of MMP12-expressing cells, skin and heart sections from PBS and Ang II infused mice were double stained for MMP-12/Mac3 and MMP-12/Collagen Type I. Macrophage infiltration was greatly increased in the heart around the injured vessels and in the interstitium (Fig. 4A), as well as in the skin (Fig. 4B). A large proportion of the Mac3-positive cells also stained for MMP-12 (Fig. 4A, B). The number of collagen-expressing cells was also greatly increased, but MMP-12 was not expressed in those cells in either the heart or the skin (Fig. 5A, B).

### Ang II induces expansion of PDGFRβ-positive cells in mouse heart and skin

**Figure 6:**
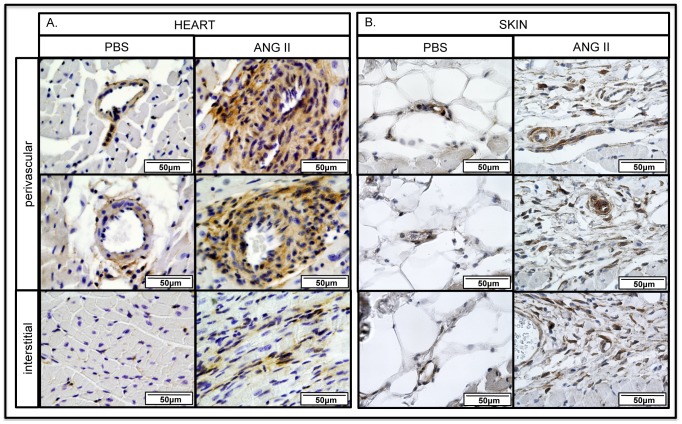
Ang II increases the number of PDGFRβ positive cells in mouse heart and skin. IHC staining of PDGFRβ was performed on paraffin sections from the heart (**A**) and skin (**B**) of PBS and Ang II treated WT mice. Representative photographs from five animals per group.

Data presented in the previous sections demonstrate that several of the characteristic features of SSc vascular injury are reproduced in the Ang II model. Since expansion of PDGFRβ-positive cells was reported in SSc [38], [39], we performed immunohistochemical staining of PDGFRβ. PDGFRβ-positive cells were primarily localized to the vessels in control mice, but were greatly increased in numbers especially in the perivascular regions, but also in the interstitial fibrotic areas in the heart of Ang-II-infused mice (Fig. 6A). The number of PDGFRβ-positive cells was also greatly increased in the fibrotic regions of the skin primarily in the lower dermis, where the fat layer was replaced with extracellular matrix (Fig. 6B).

### MMP12KO mice are protected from heart and skin fibrosis in the Ang II model

**Figure 7:**
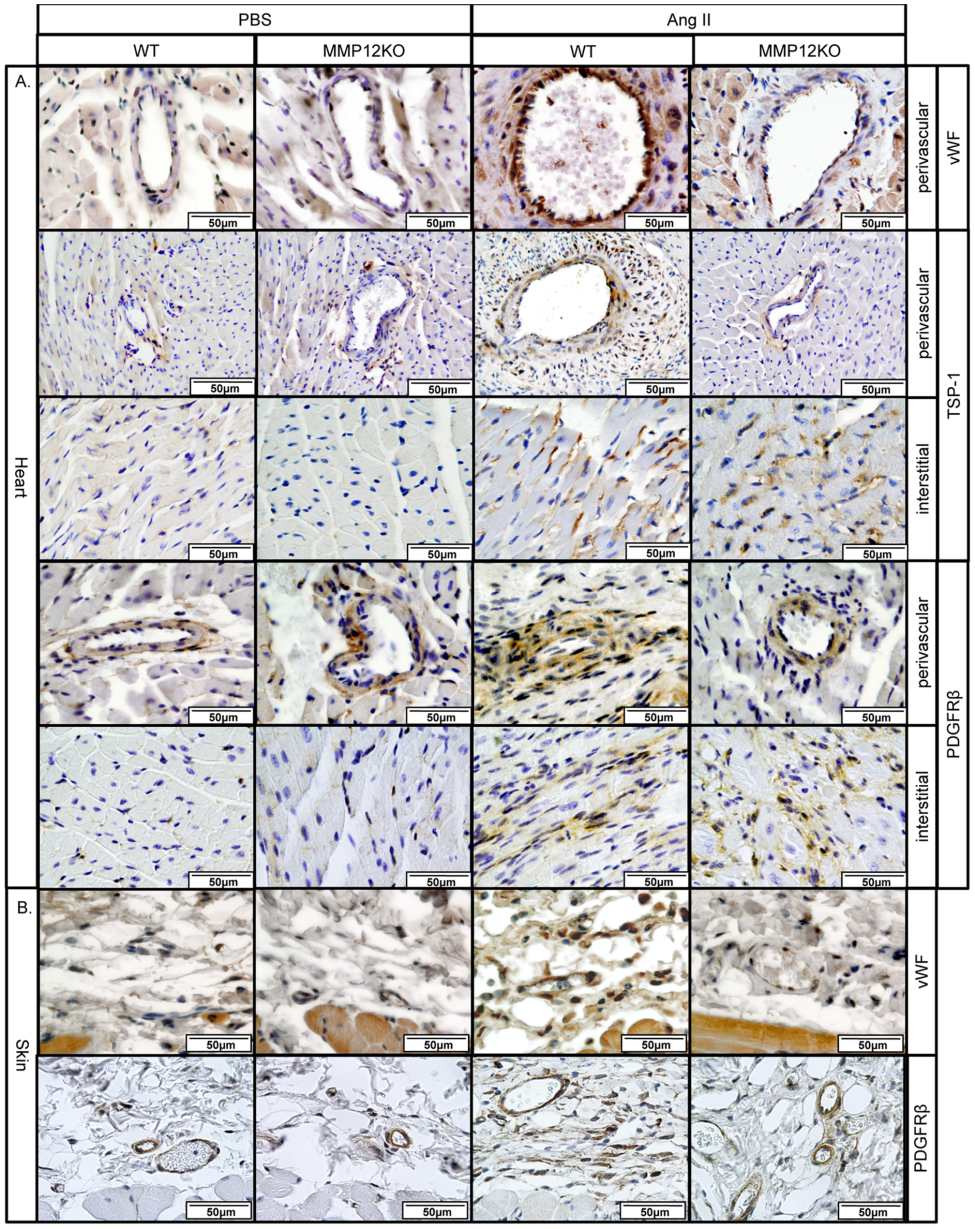
Reduced levels of vascular and pericyte markers in MMP12KO mice. **A**. IHC staining of vWF, TSP-1 and PDGFRβ was performed on paraffin sections from the heart of PBS and Ang II treated WT and MMP12KO mice. **B**. IHC staining of vWF, and PDGFRβ was performed on paraffin sections from the skin of PBS and Ang II treated WT and MMP12KO mice. Representative photographs are shown from three animals per group.

**Figure 8:**
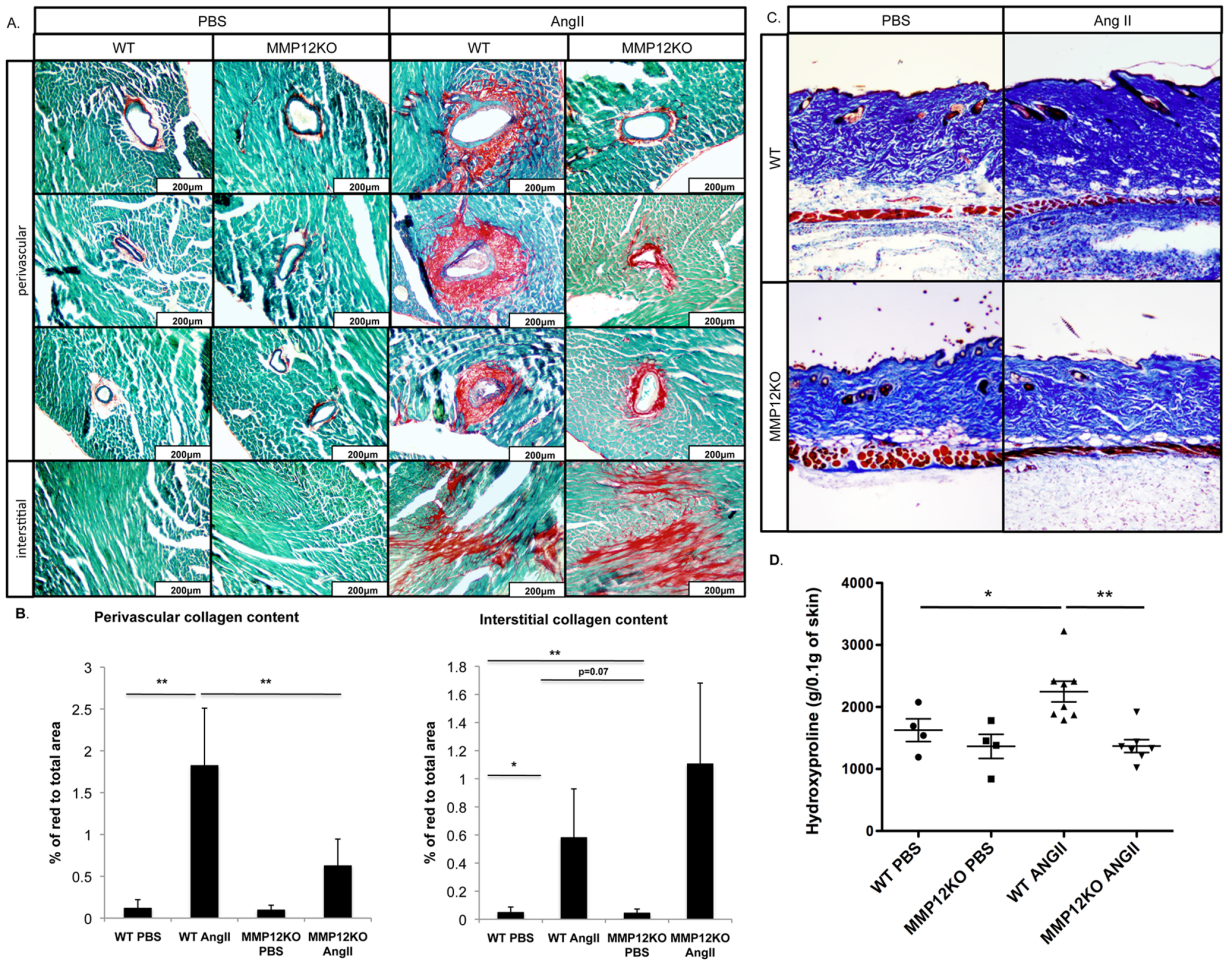
MMP12KO mice are partially protected from heart and skin fibrosis in the Ang II model. Picrosirius Red and Gomori's Trichrome staining was performed on paraffin sections respectively from the heart (**A**) and skin (**B**) of WT and MMP12KO mice infused with PBS or Ang II. Representative photographs are shown from five animals per group. **C**. Perivascular and interstitial collagen content in the heart was quantified using ImageJ software by measuring the ratio of the red area to the total area of the heart. **D**. Total hydroxyproline content in PBS- and Ang II-treated WT and MMP12KO mice. Values are the mean±SD of 6 mice in each group; *p≤0.05; **p≤0.01.

MMP12KO mice were used to assess the contribution of MMP-12 to vascular injury and fibrosis in the Ang II model. IHC staining of the perivascular regions of the heart of MMP12KO mice showed that in contrast to WT mice, only a very small increase of vWF, TSP-1, and PDGFRβ^+^ cells was observed after Ang II infusion, which was comparable to the PBS-infused mice (Fig. 7A). Likewise, vWF and PDGFRβ was not elevated in the skin of Ang II treated MMP12KO mice (Fig.7B). Unexpectedly, in the fibrotic interstitial heart regions the number of TSP-1 and PDGFRβ positive cells was not decreased in the Ang II infused MMP12KO mice and was comparable to the wild type mice. Consistent with these findings, collagen deposition in the heart, as shown by PicroSirius Red staining, was significantly reduced in the perivascular regions (Fig. 8A, B). In contrast, interstitial heart fibrosis was moderately increased in MMP12KO mice (Fig. 8A, B). Histological examination of the skin using Gomori's Trichrome staining also showed decreased collagen deposition (Fig. 8C). Moreover, total hydroxyproline content measured in the skin of Ang II-treated mice was significantly reduced in MMP12KO mice when compared to wild-type controls (Fig. 8D). Together, these data show that MMP-12 deficiency attenuates vascular injury and fibrosis in the skin and in the perivascular regions of the heart. MMP12KO mice were not protected from developing interstitial heart fibrosis suggesting a different mechanism regulating profibrotic responses in different regions of the heart in response to Ang II.

### MMP-12 deficiency inhibits Ang II-induced accumulation of Arg 1^+^ macrophages in the skin and in the perivascular regions of the heart

**Figure 9:**
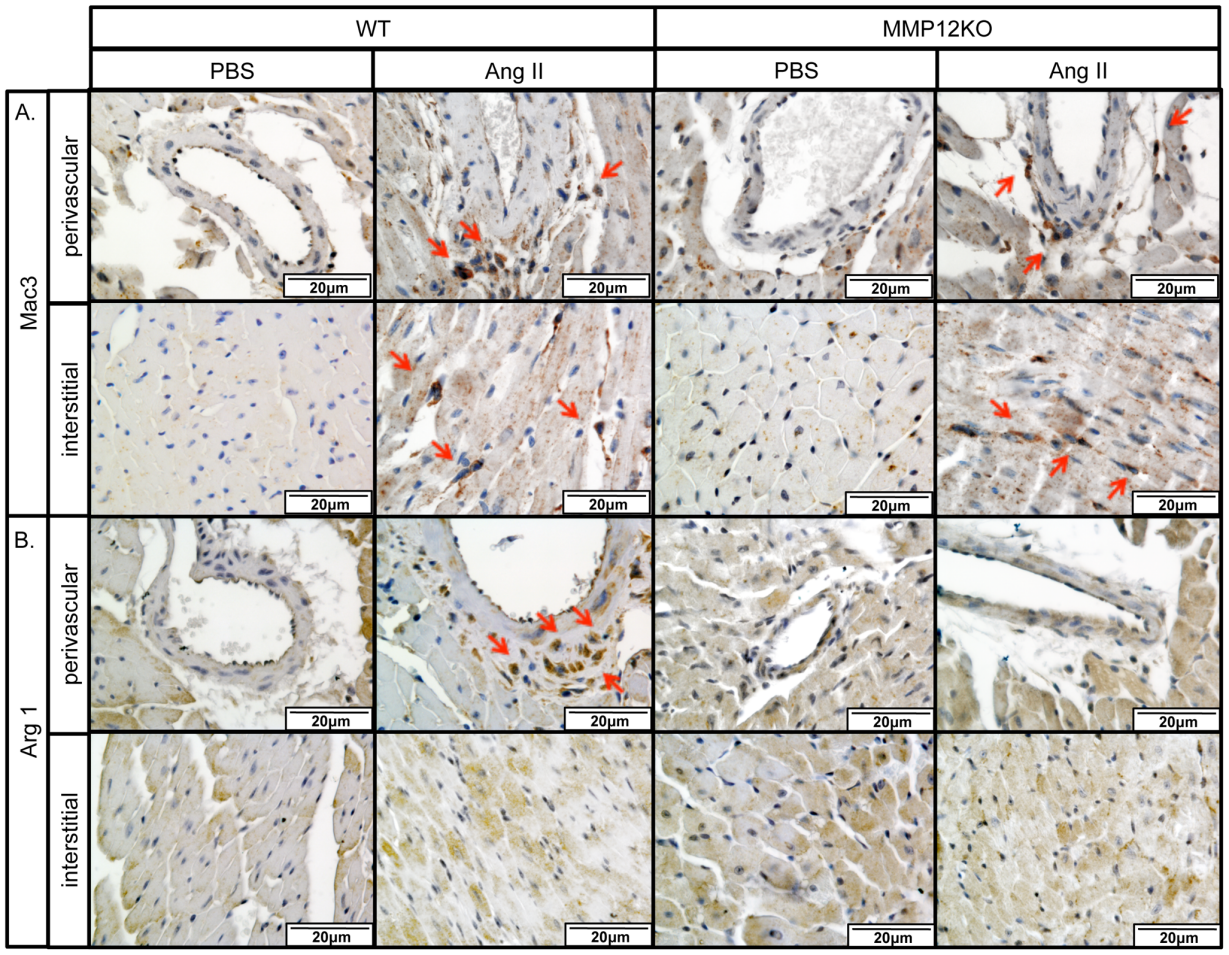
Distribution of Mac3^+^ and Arg 1^+^ macrophages in the Ang II treated mouse hearts. IHC staining of Mac3 (**A**) and Arg 1 (**B**) was performed on paraffin sections from the hearts of PBS and Ang II treated WT mice. Representative photographs are shown from four animals per group. Arrows indicate positive cells.

Macrophages are commonly found in association with fibrosis, including heart and skin [40]. To assess the effect of MMP-12 deficiency on Ang II-induced macrophage accumulation, heart and skin sections from PBS and Ang II infused mice were examined by immunohistochemical staining for a general macrophage marker, Mac3, and a representative marker of alternatively activated (M2) macrophages, Arg 1. Mac3^+^ cells were present throughout the heart in the PBS-infused mice, but after Ang II infusion their number was greatly increased in the fibrotic regions around the vessels and in the interstitium (Fig. 9A). The number of Mac3^+^ cells was largely unaffected by the MMP-12 knockout (Fig. 9A). In contrast, Arg 1 positive cells were detected in the fibrotic perivascular regions in the Ang II infused mice, but were not found in the interstitial fibrotic heart regions or in the PBS infused mice (Fig. 9B). Arg 1^+^ cells were significantly decreased in the Ang II infused MMP12KO mice (Fig. 9B).

**Figure 10:**
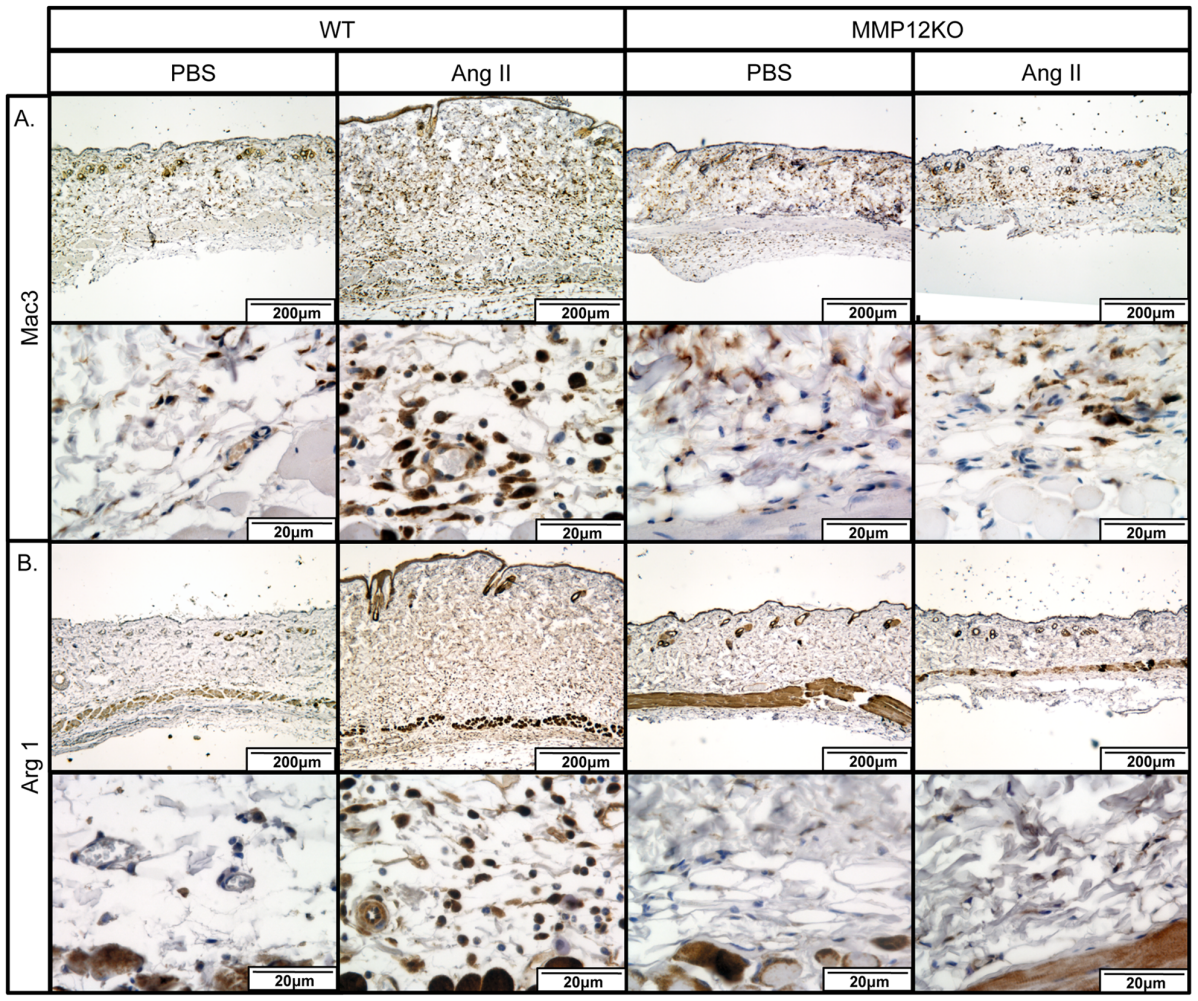
Distribution of Mac3^+^ and Arg 1^+^ macrophages in the Ang II treated mouse skin. IHC staining of Mac3 (**A**) and Arg 1 (**B**) was performed on paraffin sections from skin of PBS and Ang II treated WT mice. Representative photographs are shown from four animals per group.

In the skin of Ang II infused WT mice Mac3^+^ cells were increased, while there were fever Mac3^+^ cells found in Ang II infused MMP12KO mice (Fig. 10A). Furthermore, Arg 1^+^ cells were very rare in the PBS infused skin, but were markedly increased in the Ang II infused mice. Consistent with the heart data, Arg 1^+^ cells were significantly decreased in the Ang II infused MMP12KO mice (Fig. 10B).

### MMP-12 deficiency inhibits Ang II-induced production of profibrotic factors in the skin and in the perivascular regions of the heart

**Figure 11:**
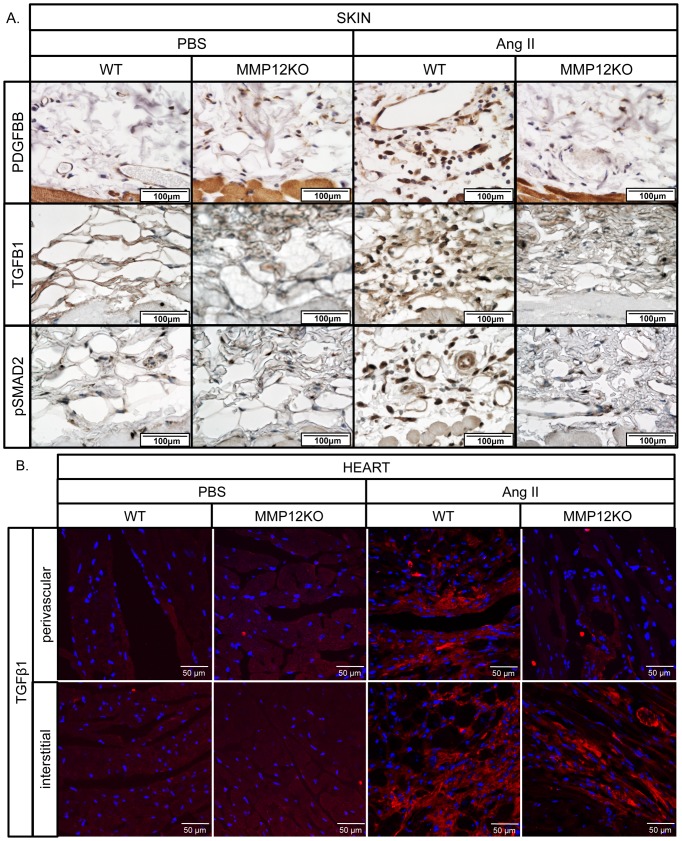
MMP-12 deficiency inhibits production of PDGFBB, TGFβ1 and pSMAD2 in the Ang II treated skin and heart. A. IHC staining of PDGFBB, TGFβ1 and pSMAd2 was performed on paraffin sections from the skin. B. Immunofluorescence staining of TGFβ1 was performed on cryosections from the heart. Representative photographs are shown from four animals per group.

To determine the effect of MMP-12 deficiency on Ang II-induced production of profibrotic mediators, heart and skin sections from PBS and Ang II infused mice were examined for PDGFBB, TGFβ1 and pSmad2 by IHC. Increased number of PDGFBB, TGFβ1 and pSmad2-positive cells were present throughout the lower dermis in the skin of Ang II-treated mice (Figure 11A), but expression of those profibrotic mediators was greatly reduced in the skin of Ang II infused MMP-12 knockout mice. Consistent with the skin data, TGFβ1 positive cells were present in the fibrotic perivascular, as well as in the interstitial fibrotic heart regions, in the Ang II infused WT mice (Fig. 11B). In contrast, in the Ang II infused MMP12KO mice, TGFB1 positive cells were significantly decreased in the perivascular regions of the heart but not in the interstitium (Fig. 11B). Because of the high muscle-specific background, PDGFBB IHC was not feasible in the heart.

## Discussion

SSc is a complex, multifactorial disease characterized by vasculopathy, immune activation and widespread tissue fibrosis. Despite intensive efforts, the molecular mechanisms responsible for the disease manifestation remain incompletely elucidated. In order to develop more effective therapies and to gain a more comprehensive insight into the pathogenesis of SSc, a better understanding of the nature of the altered cross-talk between different cell types is needed. One of the factors that impedes progress in this area is a shortage of appropriate animal models that reflect the complex nature of SSc. We have previously reported that Ang II induces dermal fibrosis accompanied by an increased number of αSMA^+^ myofibroblasts, fibrocytes, and CD163^+^ macrophages [18]. Here we demonstrate that Ang II also induces prominent vascular injury that recapitulates several aspects of SSc vasculopathy, including elevated levels of MMP-12 in the skin and in the hearts of Ang II-treated mice. Additionally, we show that mice lacking MMP-12 are protected from developing vascular injury, M2 macrophage accumulation, and dermal and perivascular heart fibrosis.

The elevated expression of vascular injury markers, including vWF and TSP-1 is well documented in SSc. Elevated serum concentration of vWF as well as its secretion into the perivascular spaces was shown to be an early marker of vascular involvement in SSc patients [3]. At the site of vascular damage vWF is rapidly released from endothelial cells and by binding to peri-endothelial collagen fibers contributes to platelet adhesion, aggregation, and thrombosis [3]. TSP-1 and -2 are secreted multifunctional matrix glycoproteins with anti-angiogenic and profibrotic properties [41]. Increased levels of TSP were reported in plasma and skin of SSc patients [4], [42], [43]. In the Ang II-treated mice, a prominent induction of vWF and TSP-1 was observed in the endothelial cells and perivascular connective tissue cells in the heart and skin, consistent with the presence of vascular injury in this model.

MMP-12 was first identified and described in mouse and human alveolar macrophages [44], [45]. MMP-12 is a zinc containing protein that is expressed as a 54 kDa inactive proenzyme that can be rapidly processed into a 22 kDa active form [27]. In addition to its matrix remodeling function, MMP-12 plays an important role in regulating the inflammatory response [46]. Furthermore, it was shown that intracellular MMP-12 can also function as a transcription regulator [47]. MMP-12 was previously indentified as a critical mediator of fibrosis in several models of tissue injury, including asthma, chronic obstructive pulmonary disease (COPD) and pulmonary fibrosis [48] [28] [49]. In line with those studies we show that MMP-12 plays a pathogenic role during development of Ang II-induced skin and perivascular heart fibrosis. On the other hand, MMP-12 deficiency appears to worsen interstitial heart fibrosis suggesting that MMP-12 may have divergent effects in different heart regions. This divergent function of MMP-12 in the perivascular and interstitial regions during Ang II-induced injury correlated with the presence M2 macrophages. Notably, activation and accumulation of M2 macrophages was shown to be associated with profibrotic changes in various models of injury including cardiac fibrosis [50], [51]. Moreover, recent reports demonstrated that M2 macrophages can produce profibrotic mediators, including TGFβ1 [52] and PDGFBB [53]. Our data indicate that accumulation of M2 macrophages occurred in the skin and in the perivascular regions of the heart in the Ang II infused wild type mice, but not in the MMP12KO mice, which might have contributed to the decreased collagen deposition in the knockout mice.

Because of the pleiotropic, not yet fully understood, nature of MMP-12, its role in response to injury may vary depending on the cellular context. For example, in the model of corneal injury, MMP12KO mice showed an exaggerated response, which was attributed to increased angiogenesis and altered chemokine production and subsequent changes in the immune response, including a decrease of neutrophil influx at day 1 and increased macrophage infiltration at day 6 [54]. It is possible that MMP-12 may play a similar role during development of interstitial fibrosis in our model. On the other hand, MMP-12 due to its matrix degrading activity could have an additional harmful effect on the large blood vessels leading to activation of adventitial cells that contribute to collagen deposition. The existence of different fibrogenic cell populations in the adventitia and interstitium have been previously described [55], which may underlie this differential response to MMP-12 deficiency. Further studies are warranted to dissect these specific mechanisms. Importantly, MMP12KO mice also showed greatly reduced markers of vascular injury such as TSP-1 and vWF, thus underscoring a central role of MMP-12 in vessel damage in the Ang II model. It is tempting to speculate that in this model fibrosis develops as a consequence of vascular injury, possibly through the generation of profibrotic growth factors and cytokines by the injured endothelial cells [35]. Future studies will be directed at answering these important questions.

In conclusion, we report that the Ang II model complements previously described genetic models of SSc [56], [57] in recapitulating several key pathogenic features of the disease including skin fibrosis, endothelial cell injury, and inflammation. This inducible model of SSc should be very useful in further elucidating the mechanisms governing SSc and in dissecting the cross-talk between different cell types contributing to the development of fibrosis. In addition, it will permit the evaluation of the effects of therapeutic treatments on various disease aspects, including vasculopathy and fibrosis. This study also demonstrates that upregulated expression levels of MMP-12 contributes to vessel injury and fibrosis in the skin and suggests that MMP-12 may represent an attractive therapeutic target for SSc.

## Supporting Information

Figure S1 **Ang II increases number of apoptotic cells in mouse heart and skin.** IHC staining of Cleaved Caspase 3 was performed on paraffin sections from the hearts (**A**) and skin (**B**) of PBS and Ang II treated WT mice. Representative photographs are shown from four animals per group. Arrows indicate positive cells.
(TIF)
