# Supplementary figures and images for: MMP-12 Deficiency Attenuates Angiotensin II-Induced Vascular Injury, M2 Macrophage Accumulation, and Skin and Heart Fibrosis
Source: PLoS One. 2014 Oct 10;9(10):e109763. doi: 10.1371/journal.pone.0109763 (PMC4193823; doi:10.1371/journal.pone.0109763)

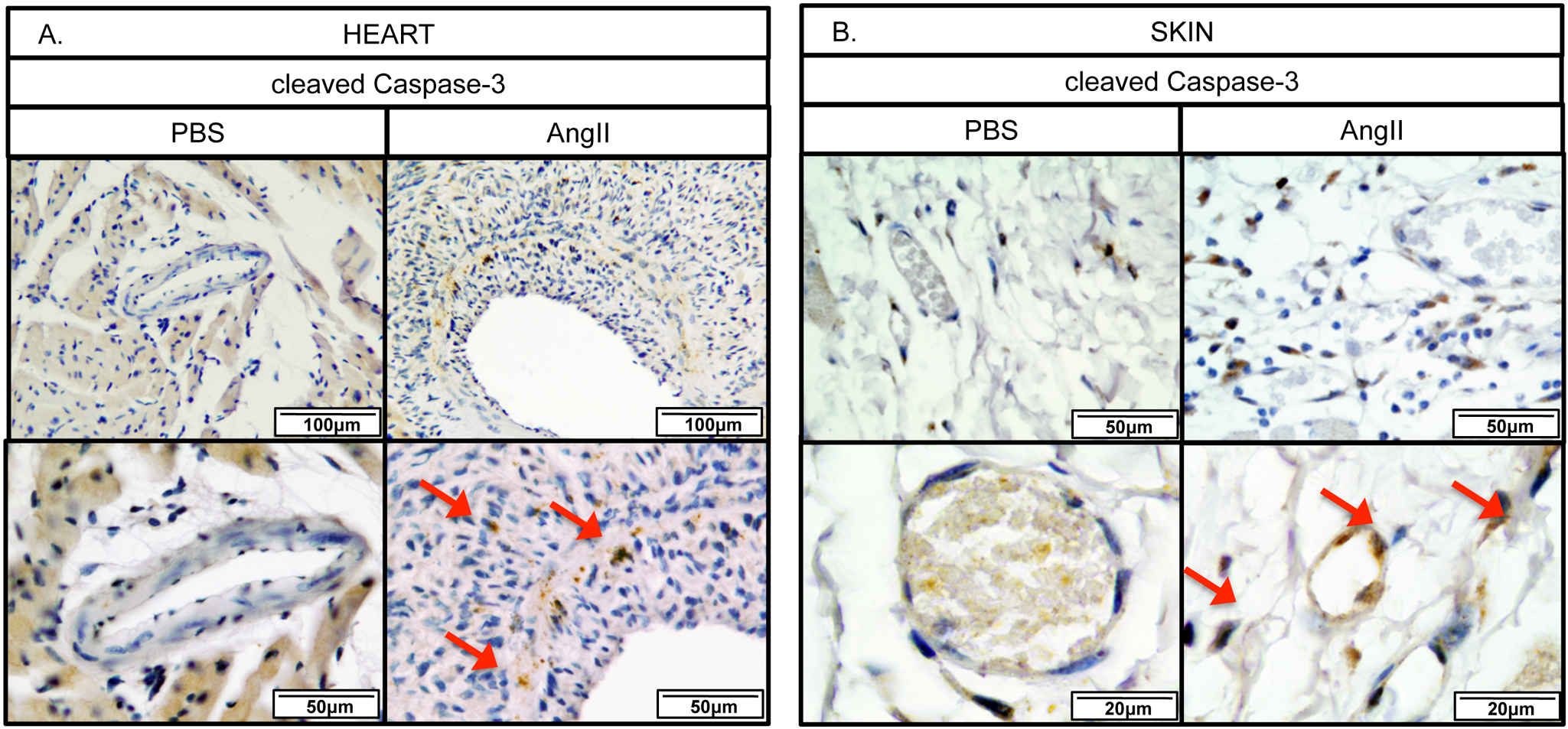

Supplement: Figure S1 — Ang II increases number of apoptotic cells in mouse heart and skin. IHC staining of Cleaved Caspase 3 was performed on paraffin sections from the hearts (A) and skin (B) of PBS and Ang II treated WT mice. Representative photographs are shown from four animals per group. Arrows indicate positive cells. (TIF) [file pone.0109763.s001.tif]
